# Mild hypothermia alone or in combination with anesthetic post-conditioning reduces expression of inflammatory cytokines in the cerebral cortex of pigs after cardiopulmonary resuscitation

**DOI:** 10.1186/cc8879

**Published:** 2010-02-16

**Authors:** Patrick Meybohm, Matthias Gruenewald, Kai D Zacharowski, Martin Albrecht, Ralph Lucius, Nikola Fösel, Johannes Hensler, Karina Zitta, Berthold Bein

**Affiliations:** 1 Department of Anaesthesiology and Intensive Care Medicine, University Hospital Schleswig-Holstein, Campus Kiel, Schwanenweg 21, Kiel, 24105, Germany; 2 Clinic of Anaesthesiology, Intensive Care Medicine and Pain Therapy, University Hospital Frankfurt, Theodor-Stern-Kai 7, Frankfurt am Main, 60590, Germany; 3 Institute of Anatomy, Christian-Albrechts-University of Kiel, Otto-Hahn-Platz 8, Kiel, 24118, Germany

## Abstract

**Introduction:**

Hypothermia improves survival and neurological recovery after cardiac arrest. Pro-inflammatory cytokines have been implicated in focal cerebral ischemia/reperfusion injury. It is unknown whether cardiac arrest also triggers the release of cerebral inflammatory molecules, and whether therapeutic hypothermia alters this inflammatory response. This study sought to examine whether hypothermia or the combination of hypothermia with anesthetic post-conditioning with sevoflurane affect cerebral inflammatory response after cardiopulmonary resuscitation.

**Methods:**

Thirty pigs (28 to 34 kg) were subjected to cardiac arrest following temporary coronary artery occlusion. After seven minutes of ventricular fibrillation and two minutes of basic life support, advanced cardiac life support was started according to the current American Heart Association guidelines. Return of spontaneous circulation was achieved in 21 animals who were randomized to either normothermia at 38°C, hypothermia at 33°C or hypothermia at 33°C combined with sevoflurane (each group: n = 7) for 24 hours. The effects of hypothermia and the combination of hypothermia with sevoflurane on cerebral inflammatory response after cardiopulmonary resuscitation were studied using tissue samples from the cerebral cortex of pigs euthanized after 24 hours and employing quantitative RT-PCR and ELISA techniques.

**Results:**

Global cerebral ischemia following resuscitation resulted in significant upregulation of cerebral tissue inflammatory cytokine mRNA expression (mean ± SD; interleukin (IL)-1β 8.7 ± 4.0, IL-6 4.3 ± 2.6, IL-10 2.5 ± 1.6, tumor necrosis factor (TNF)α 2.8 ± 1.8, intercellular adhesion molecule-1 (ICAM-1) 4.0 ± 1.9-fold compared with sham control) and IL-1β protein concentration (1.9 ± 0.6-fold compared with sham control). Hypothermia was associated with a significant (*P *< 0.05 versus normothermia) reduction in cerebral inflammatory cytokine mRNA expression (IL-1β 1.7 ± 1.0, IL-6 2.2 ± 1.1, IL-10 0.8 ± 0.4, TNFα 1.1 ± 0.6, ICAM-1 1.9 ± 0.7-fold compared with sham control). These results were also confirmed for IL-1β on protein level. Experimental settings employing hypothermia in combination with sevoflurane showed that the volatile anesthetic did not confer additional anti-inflammatory effects compared with hypothermia alone.

**Conclusions:**

Mild therapeutic hypothermia resulted in decreased expression of typical cerebral inflammatory mediators after cardiopulmonary resuscitation. This may confer, at least in part, neuroprotection following global cerebral ischemia and resuscitation.

## Introduction

Although initial return of spontaneous circulation (ROSC) from cardiac arrest is achieved in about 30 to 40% of cases, only 10 to 30% of the patients admitted to the hospital will be discharged with good outcome [1]. One third of those who survive, suffer persistent neurological impairments [2]. Mild therapeutic hypothermia has emerged as the most effective strategy to reduce neurological impairment after successful cardiopulmonary resuscitation (CPR) [3]. The precise mechanisms by which mild hypothermia protects brain cells remain to be elucidated, but it is very likely that hypothermia acts upon multiple pathways including reduction in cerebral metabolism and oxygen consumption, attenuation of neuronal damage, and inhibition of excitatory neurotransmitter release [4].

There is growing evidence on the damaging nature of the inflammatory response following brain ischemia. Various inflammatory cytokines have been implicated as important mediators of ischemia/reperfusion injury following both focal and global cerebral ischemia [5]. Most of the previous experimental studies induced global cerebral ischemia by bilateral carotid artery occlusion as a correlate of cardiac arrest, but inflammatory response mechanisms following carotid artery occlusion and anti-inflammatory mechanisms of hypothermia may be different from those observed after cardiac arrest and manual CPR. Thus, it is unknown whether cardiac arrest also triggers the release of cerebral inflammatory molecules, and whether therapeutic hypothermia alters this inflammatory response.

Neuronal injury may also result in necrotic and apoptotic cell death. In contrast to necrosis (cell death by acute injury), apoptosis is a well-regulated physiological process. Cells undergoing apoptosis are characterized by cytoplasmic shrinkage, nuclear condensation, and formation of membrane-bound vesicles. Key elements of the apoptotic pathway include changes in the gene expression of the pro-apoptotic protein Bax and the apoptosis-suppressing protein Bcl-2. The extent to which hypothermia affects cerebral apoptosis-related proteins after successful CPR is not clear [4].

The mismatch between early survival and final outcome after CPR emphasizes the importance of further research on potential adjuvants in addition to mild hypothermia. Specifically, pharmacological post-conditioning may offer an attractive opportunity to further ameliorate damage to the brain in the post-resuscitation period. While the volatile anesthetic sevoflurane has emerged as a pre-conditioning-like agent with significant neuroprotective effects in models of focal and global cerebral ischemia [6], its potential neuroprotective and anti-inflammatory properties have not yet been investigated in the context of post-resuscitation care. Thus, a combination of hypothermia and anesthetic post-conditioning with sevoflurane may extend neuroprotection, as it has recently been shown for the noble anesthetic gas xenon combined with hypothermia after neonatal hypoxia-ischemia [7].

We hypothesized that hypothermia attenuates cerebral inflammatory response in a pig model of global cerebral ischemia following cardiac arrest. We further hypothesized that the volatile anesthetic sevoflurane, when administered during reperfusion after successful CPR, confers additional anti-inflammatory effects.

## Materials and methods

The project was approved by the Animal Investigation Committee of the University Schleswig-Holstein, Campus Kiel, Germany, and animals were managed in accordance with the guidelines of the University Schleswig-Holstein, Campus Kiel, Germany, and the Utstein-style guidelines [8]. All animals received human care in compliance with the *Guide for the Care and Use of Laboratory Animals *published by the National Institute of Health (NIH Publication No. 88.23, revised 1996).

### Animals

This is an experimental study on 40 healthy pigs (cardiac arrest: n = 30; sham control: n = 5; excluded from study n = 5) aged three to four months of both gender, weighing 28 to 34 kg. Anesthesia was initiated by intramuscular injection of 8 mg/kg azaperone and 0.05 mg/kg atropine, and completed by intravenous injection of 1 to 2 mg/kg propofol and 0.3 μg/kg sufentanil. After endotracheal intubation, pigs were ventilated with a volume-controlled ventilator (Draeger, Sulla 808V, Luebeck, Germany) and the following setting: a FiO_2 _of 0.3 at 20 breaths/minute, a tidal volume of 8 mL/kg to maintain normocapnia, and a positive end-expiratory pressure of 5 mm Hg. Ventilation was monitored using an inspired/expired gas analyzer that measured oxygen and end-tidal carbon dioxide (suction rate, 200 mL/min; M-PRESTN; Datex-Ohmeda Inc., Helsinki, Finland). Total intravenous anesthesia (TIVA) was maintained by continuous infusion of 4 to 8 mg/kg/h propofol and 0.3 μg/kg/h sufentanil; muscle relaxation was achieved by continuous infusion of 0.2 mg/kg/h pancuronium. Depth of anesthesia was judged according to blood pressure, heart rate and Bispectral Index (BISXP, Aspect Medical Systems, Natick, MA, USA) [9]. In order to assure an appropriate depth of anesthesia we performed also indirect measures such as tail clamping, monitoring of the corneal reflex and lacrimation, as well as changes in hemodynamics and heart rate. If assessment suggested inadequate level of anesthesia, additional sufentanil and propofol was injected. Ringer's solution was administered continuously throughout the preparation phase to replace fluid loss during instrumentation. Standard leads II and V_5 _electrocardiogram were used to monitor cardiac rhythm.

A 7F saline-filled central venous catheter was inserted in the right internal jugular vein for drug administration. A 4F thermistor-tipped catheter for arterial thermodilution (Pulsion Medical Systems, Munich, Germany) was inserted percutaneously into the right femoral artery. The arterial catheter was connected to the PiCCO system (PiCCO plus, Software Version 6.0, Pulsion Systems, Munich, Germany), and the resulting signal processed to determine mean arterial blood pressure, heart rate, and blood temperature. In addition, the arterial catheter allowed discontinuous measurement of transpulmonary cardiac output by injecting 10 mL ice cold saline into the proximal port of the central venous catheter. The mean of three consecutive measurements randomly assigned to the respiratory cycle was used for determination of cardiac output. Cardiac index was calculated as the ratio of cardiac output/body surface area (body surface area = 0.0734*(body weight in kg)^0.656 ^[10]). Intravascular catheters were attached to pressure transducers (Smiths Medical, Kirchseeon, Germany) that were aligned at the level of the right atrium.

### Experimental protocol

**Figure 1:**
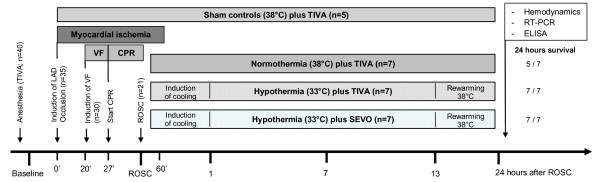
**Experimental time line**. Thirty pigs were subjected to cardiac arrest following left anterior descending (LAD) coronary artery ischemia. Ventricular fibrillation (VF) was electrically induced twenty minutes after LAD occlusion. After seven minutes of VF, pigs were resuscitated (CPR). After successful return of spontaneous circulation (ROSC; n = 21), coronary perfusion was reestablished after 60 minutes of LAD occlusion, and animals were randomized either to normothermia at 38°C, hypothermia at 33°C or hypothermia at 33°C combined with sevoflurane (each group n = 7) for 24 hours. Five animals were sham operated. In the normothermia group, five out of seven animals survived for 24 hours compared to all animals in the hypothermia and hypothermia combined with sevoflurane group.

The experimental time line is presented in Figure 1. Because the majority of patients experience cardiac arrest due to myocardial ischemia [11], and because this scenario has only been considered in few animal experiments, our study is based on an experimental porcine model of cardiac arrest following acute coronary artery ischemia reflecting a realistic clinical setting. Five healthy animals served as sham controls, which were anesthetized with TIVA until the end of the experiment. Thirty-five pigs underwent left anterior descending (LAD) coronary artery occlusion for 60 minutes according to the technique previously described [12]. Five pigs fibrillated spontaneously following left anterior descending coronary artery occlusion, which were excluded from further analysis. Thirty pigs were then subjected to cardiac arrest 20 minutes after LAD occlusion. Ventricular fibrillation was electrically-induced by an alternating current of 5 to 10 V in a standardized manner, and mechanical ventilation was discontinued. After a seven-minute non-intervention interval of untreated ventricular fibrillation, basic life support CPR was simulated for two minutes applying external manual closed chest compressions at a rate of 100 per minute, and a compression-to-ventilation ratio of 30:2. Subsequently, advanced cardiac life support was started with 100 J biphasic defibrillation attempt (M-Series Defibrillators, Zoll Medical Corporation, Chelmsford, Massachusetts, USA), all subsequent attempts were performed with 150 J every two minutes. Ventilations were performed with 100% oxygen at 20 breaths/minute. All pigs received 45 μg/kg epinephrine and 0.4 U/kg vasopressin alternating as suggested by the American Heart Association guidelines [13]. ROSC was defined as maintenance of an unassisted pulse and a systolic aortic blood pressure of ≥60 mm Hg lasting for 10 consecutive minutes according to the Utstein-style guidelines [8]. Since neurological recovery is very unlikely after 30 minutes of normothermic cardiac arrest, CPR was terminated, when resuscitation remained unsuccessful after 23 minutes of CPR. After ROSC, animals were randomized either to normothermia (38°C) plus TIVA (NT), hypothermia (33°C) plus TIVA (HT), or hypothermia (33°C) combined with 2.0 Vol% end-tidal sevoflurane and 0.3 μg/kg/h sufentanil (HT+SEV). Since hypothermia was shown to increase blood concentrations of propofol by about 30% [14], we reduced continuous infusion of propofol during hypothermia targeting bispectral index values below 60. Body core temperature was monitored continuously by the arterial catheter, and normothermic body temperature was maintained at 38.0°C with a heating blanket, since the physiological rectal temperature of pigs is supposed to be about 38°C [15]. Hypothermia was induced by 1,000 mL saline (4°C) and maintained by a cooling device (Icy catheter and CoolGard 3000; Alsius Corp, Irvine, CA, USA) that was introduced into the femoral vein. According to the landmark study by Bernard et al. [16] we used a target body temperature of 33°C for 12 hours. Thereafter, re-warming was initiated (0.5°C per hour). One hour after ROSC, FiO_2 _was reduced to 0.4. During the post-resuscitation period, animals received crystalloid infusions to keep central venous pressure above 8 mm Hg and mean arterial blood pressure above 50 mm Hg. If this first step failed, additional norepinephrine was administered to keep mean arterial blood pressure above 50 mm Hg. We further aimed at serum glucose levels less than 150 mg/dL by intermittent insulin bolus administration. Animals were killed by an overdose of sufentanil, propofol and potassium chloride 24 hours after ROSC. Tissue samples of the cerebral cortex were collected within 15 seconds following euthanasia *via *a craniotomy that was established before euthanasia, and then immediately snap-frozen in liquid nitrogen (stored at -80°C) to minimize time-dependent effects of cerebral ischemia following euthanasia on cytokine expression. Autopsy was routinely performed for documentation of potential injuries to the thoracic and abdominal cavity during CPR.

Hemodynamic data, including mean arterial blood pressure, heart rate, end-tidal carbon dioxide, and cardiac index were determined at baseline (BL), following ROSC, and 7 and 24 hours after ROSC, respectively.

### Quantitative real-time RT-PCR

Transcript levels of interleukin (IL)-1β, IL-6, IL-10, tumor necrosis factor (TNF)α, intercellular adhesion molecule (ICAM)-1, and the apoptosis-associated proteins Bcl-2 and Bax were investigated in the cerebral cortex tissue of all surviving animals and compared with tissue of sham control animals. Tissue samples were analyzed by a person blinded to treatment assignment. Fully detailed description of quantitative real-time RT-PCR is presented in the Additional File 1 and Table S1 [17-20].

### Enzyme-linked immunosorbent assay (ELISA)

Protein concentrations of IL-1β were determined by a swine specific ELISA (BioSource International, Inc. Camarillo, CA, USA) in homogenates of frozen tissues according to the manufacturer's protocol. All ELISA assays were carried out in duplicates.

### Statistical analysis

Statistics were performed using commercially available statistics software (GraphPad Prism version 5.02 for Windows, GraphPad Software, San Diego, CA, USA). Survival rates were compared using Fisher's exact test. Statistical analysis was performed with a one-way analysis of variance (ANOVA) followed by a Bonferroni post hoc test to correct for multiple measurements. RT-PCR data analysis was performed according to a relative standard curve method using an Excel spreadsheet, and statistical significance was tested using two-sided Pair-wise fixed Reallocation Randomisation Test, as provided in the REST2005 program [20]. The Mann-Whitney test was used for analysis of protein concentrations of IL-1β where normal distribution was not expected. Variables are expressed as mean ± SD unless otherwise specified. Statistical significance was considered at a two-sided *P *value of ≤ 0.05.

## Results

### Cardio-pulmonary resuscitation

Twenty-one animals were successfully resuscitated. Detailed resuscitation data are presented in Table 1. In the NT group, five out of seven animals survived for 24 hours compared to all animals in the HT and HT+SEV group (*P *= 0.46 vs. NT). Two animals of the NT group died due to hemodynamic instability during the post-resuscitation period.

**Table 1:** Cardiopulmonary resuscitation data

	NT	HT	HT+SEV	*P *values
ROSC rate [n]	7/10	7/10	7/10	-
CPR time to successful resuscitation [min]	9.7 ± 2.8	10.3 ± 3.4	10.5 ± 3.1	0.939
Cumulative epinephrine dose [μg/kg]	100 ± 44	101 ± 47	93 ± 33	0.828
Cumulative vasopressin dose [IU/kg]	0.8 ± 0.2	0.8 ± 0.3	0.8 ± 0.3	0.897
Cumulative defibrillation energy [J]	755 ± 420	703 ± 413	795 ± 199	0.854
CorPP 10 [mm Hg]	31 ± 9	26 ± 11	28 ± 6	0.559
CorPP 15 [mm Hg]	39 ± 28	40 ± 25	38 ± 24	0.890
Time to target temperature 33°C [min]	-	47 ± 10	45 ± 15	-

ROSC, return of spontaneous circulation; CPR, cardiopulmonary resuscitation. Time to successful resuscitation, cumulative epinephrine and vasopressin dose, cumulative defibrillation energy, coronary perfusion pressure (CorPP) 10 and 15 minutes after induction of ventricular fibrillation, and induction time to target temperature of 33°C. NT indicates normothermia; HT, hypothermia; HT+SEV, hypothermia combined with sevoflurane. Data are mean ± SD.

### Post-resuscitation hemodynamics

Post-resuscitation systemic hemodynamic variables are presented in Table 2. Heart rate, mean arterial blood pressure and cardiac index did not significantly differ between groups. Cumulative crystalloid fluid load and cumulative norepinephrine doses were not significantly different between groups 24 hours after ROSC (volume load (*P *= 0.540), norepinephrine doses (*P *= 0.812); NT: 4241 ± 1244 mL, 4.4 ± 1.6 mg; HT: 3987 ± 932 mL, 4.9 ± 2.1 mg; HT+SEV: 4627 ± 1056 mL, 5.1 ± 1.8 mg).

**Table 2:** Hemodynamic data

	NT	HT	HT+SEV	*P* value
**Baseline**				
HR, beats/minute	107 ± 21	105 ± 14	96 ± 15	0.342
MAP, mm Hg	65 ± 13	70 ± 11	71 ± 13	0.314
ETCO_2_, mm Hg	40 ± 5	36 ± 4	37 ± 5	0.180
CI, L/min/m^2^	7.4 ± 1.8	6.8 ± 1.3	7.4 ± 1.7	0.580
**ROSC**				
HR, beats/minute	94 ± 18	99 ± 33	96 ± 21	0.988
MAP, mm Hg	55 ± 6	65 ± 21	60 ± 8	0.225
ETCO_2_, mm Hg	39 ± 9	42 ± 4	39 ± 7	0.363
CI, L/min/m^2^	4.4 ± 0.5	4.5 ± 1.9	4.6 ± 1.0	0.982
**Seven hours ROSC**				
HR, beats/minute	131 ± 17	140 ± 22	127 ± 20	0.821
MAP, mm Hg	58 ± 4	59 ± 12	61 ± 8	0.820
ETCO_2_, mm Hg	37 ± 7	35 ± 4	35 ± 2	0.731
CI, L/min/m^2^	6.2 ± 0.4	5.5 ± 1.1	7.1 ± 1.2	0.071
**24 hours ROSC**				
HR, beats/minute	154 ± 25	143 ± 19	124 ± 24	0.297
MAP, mm Hg	46 ± 6	57 ± 12	54 ± 4	0.249
ETCO_2_, mm Hg	37 ± 4	41 ± 1	37 ± 2	0.180
CI, L/min/m^2^	5.6 ± 0.2	7.1 ± 1.6	8.7 ± 1.8	0.139

Hemodynamic data were determined at baseline, following return of spontaneous circulation (ROSC), and 7 and 24 hours after ROSC. HR indicates heart rate; MAP, mean arterial blood pressure; ETCO_2_, end-tidal carbon dioxide, CI, cardiac index; NT, normothermia; HT, hypothermia; HT+SEV, hypothermia combined with sevoflurane. Data are mean ± SD.

### Cerebral inflammatory response

**Figure 2:**
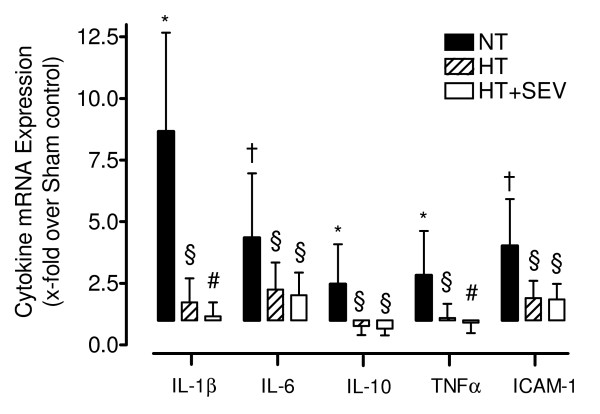
**Cerebral cytokine mRNA expression**. Transcript levels of the cerebral cytokines interleukin (IL)-1β, IL-6, IL-10, tumor necrosis factor (TNF)α and intercellular adhesion molecule (ICAM)-1 were determined by quantitative RT-PCR. NT, normothermia; HT, hypothermia; HT+SEV, hypothermia combined with sevoflurane. Data are expressed as mean ± SD (x-fold upregulation compared with Sham control). * *P *< 0.05, † *P *< 0.01 vs. Sham. §*P *< 0.05, #P < 0.01 vs. NT. RT-PCR data analysis was performed using two-sided *Pair-wise fixed Reallocation Randomisation Test*.

**Figure 3:**
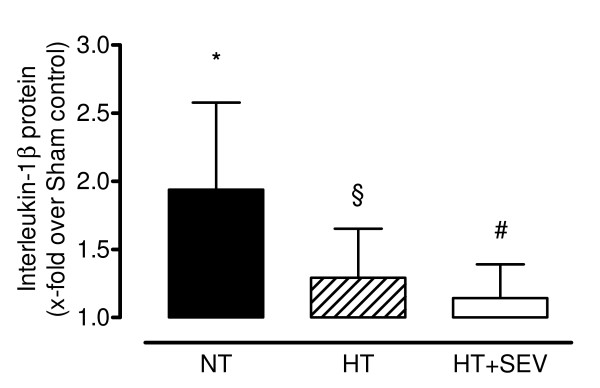
**Protein concentration of interleukin-1β**. Protein concentration of interleukin (IL)-1β was determined by a swine specific enzyme-linked-immunosorbent assay. NT, normothermia; HT, hypothermia; HT+SEV, hypothermia combined with sevoflurane. Data are expressed as mean ± SD (x-fold upregulation compared with Sham control). *P < 0.05 vs. Sham. §*P *< 0.05, #P < 0.01 vs. NT (using Mann-Whitney test).

Global cerebral ischemia following resuscitation resulted in a significant upregulation of cerebral tissue inflammatory cytokine mRNA expression (NT: IL-1β 8.7 ± 4.0, IL-6 4.3 ± 2.6, IL-10 2.5 ± 1.6, TNFα 2.8 ± 1.8, ICAM-1 4.0 ± 1.9-fold compared with sham control) and IL-1β protein concentration (1.9 ± 0.6-fold compared with sham control). Hypothermia was associated with significantly (*P *< 0.05 versus normothermia) less upregulation of mRNA expression (IL-1β 1.7 ± 1.0, IL-6 2.2 ± 1.1, IL-10 0.8 ± 0.4, TNFα 1.1 ± 0.6, ICAM-1 1.9 ± 0.7-fold compared with sham control) and IL-1β protein concentration (1.3 ± 0.4-fold compared with sham control). Sevoflurane did not confer statistically significant (versus hypothermia) additional protective effects neither on mRNA (IL-1β 1.2 ± 0.6, IL-6 2.0 ± 0.9, IL-10 0.7 ± 0.3, TNFα 0.9 ± 0.4, ICAM-1 1.8 ± 0.6-fold compared with sham control) nor on protein levels (1.1 ± 0.2-fold compared with sham control; Figures 2 and 3).

### Bax and Bcl-2 mRNA expression

**Figure 4:**
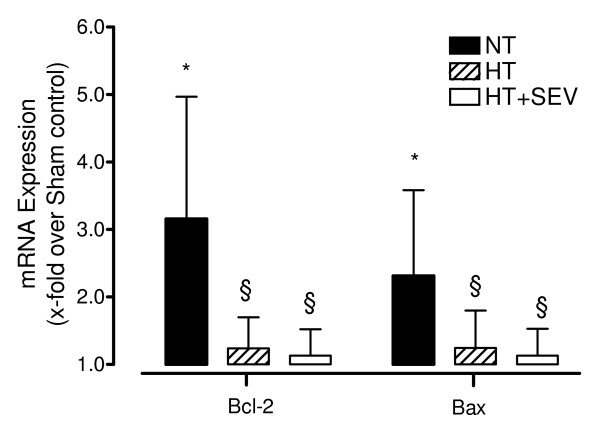
**Cerebral Bcl-2 and Bax mRNA expression**. Transcript levels of the cerebral apoptosis-associated proteins Bcl-2 and Bax were determined by quantitative RT-PCR. NT, normothermia; HT, hypothermia; HT+SEV, hypothermia combined with sevoflurane. Data are expressed as mean ± SD (x-fold upregulation compared with Sham control). *P < 0.05 vs. Sham. §*P *< 0.05 vs. NT. RT-PCR data analysis was performed using two-sided *Pair-wise fixed Reallocation Randomisation Test*.

We found a significant (*P *< 0.01) upregulation of both Bcl-2 mRNA and Bax expression after global cerebral ischemia (NT: Bcl-2 3.2 ± 1.8-fold, Bax 2.3 ± 1.3-fold compared with sham control). Hypothermia was associated with significantly (*P *< 0.05) less upregulation of mRNA expression (Bcl-2 1.2 ± 0.5-fold, Bax 1.2 ± 0.6-fold compared with sham control). Sevoflurane did not confer additional effects (Bcl-2 1.1 ± 0.4-fold, Bax 1.1 ± 0.4-fold compared with sham control; Figure 4).

## Discussion

Neurological dysfunction resulting from cardiac arrest largely contributes to morbidity and mortality after initially successful CPR [21]. Employing a pig model we showed that (i) global cerebral ischemia following cardiac arrest and CPR results in upregulation of pro-inflammatory cytokine expression in the cerebral tissue, ii) mild hypothermia significantly reduces cerebral tissue inflammatory response, and (iii) pharmacological post-conditioning with sevoflurane does not confer additional anti-inflammatory effects on cerebral tissue.

### Cerebral inflammatory response following resuscitation

Mechanisms of brain injury following cerebral ischemia are complex with multiple modulators, signaling pathways, proteins and enzymes being involved that may facilitate cell death or survival [22]. Post-ischemic inflammation has been shown to play a critical role in cerebral ischemia/reperfusion injury [23]. Specifically, there is strong evidence suggesting that a disproportionate and persistent production of cytokines can significantly increase the risk and extent of brain injury [5,24]. In terms of a systemic inflammatory response, increased serum levels of different cytokines and chemokines have recently been presented in a rat model of cardiac arrest [25], and in patients successfully resuscitated from out-of-hospital cardiac arrest [26,27]. The role of the cerebral inflammatory response after cardiac arrest, however, has poorly been investigated. Most of the previous experimental studies induced global cerebral ischemia by bilateral carotid artery occlusion as a surrogate of cardiac arrest, but inflammatory response mechanisms following carotid artery occlusion and anti-inflammatory mechanisms of hypothermia are different from the ones observed after cardiac arrest and resuscitation [27]. Youngquist et al. [28] have recently shown increased TNFα and IL-6 protein concentration in the cerebrospinal fluid following cardiac arrest. Since the presence of a lesion pattern of cortical involvement, termed as *extensive cortical lesion pattern *in MR imaging, has very recently been shown to be a very good predictor of poor neurologic prognosis after cardiac arrest [29], we focused on neuroinflammation in the cerebral cortex tissue. In our study, global cerebral ischemia following cardiac arrest resulted in a significant upregulation of mRNA expression of several cytokines in the cerebral cortex tissue. In addition, we observed a significant rise in IL-1β protein concentration in the cerebral cortex tissue that may be most probably due to local synthesis primarily by microglial cells, astrocytes and/or endothelial cells [30] rather than transport across the blood-brain barrier. This is emphasized by the data of Mizushima and colleagues who demonstrated that the integrity of the blood-spinal cord and blood-brain barriers to radiolabelled TNFα remains intact following resuscitation in a mouse model of cardiac arrest [31].

### Effects of hypothermia on cerebral inflammatory response

Several mechanisms by which hypothermia exerts its protective effects have been characterized, including reduction in excitotoxin accumulation and inhibition of molecular pathways such as apoptosis and necrosis [4]. The role of inflammation in global cerebral ischemia induced by bilateral carotid artery occlusion and focal cerebral ischemia has extensively been studied, but effects of hypothermia on global cerebral ischemia/reperfusion injury following cardiac arrest has been investigated to a much lesser extent. Webster et al. have previously found that mild hypothermia attenuated microglial activation and nuclear translocation of NFκB, and thereby reduced activation of the downstream inflammatory pathway [32]. Considering the relatively late onset of the inflammatory response and the prolonged destructive process following cerebral ischemia/reperfusion, there appears to be a reasonable therapeutic time window using mild hypothermia to favourably affect the inflammatory pathway [33]. To date, the majority of publications may suggest that hypothermia simply blocks any ischemia-induced damaging cascade. However, contrary to this popular belief, the expression of certain beneficial genes is actually upregulated by mild hypothermia [4]. Hicks and colleagues [34] further demonstrated that prolonged hypothermia during later reperfusion improved neurological outcome after experimental global ischemia and was associated with selective changes in the pattern of stress-induced protein expression. From our data we conclude that mild hypothermia initiated after successful resuscitation from cardiac arrest reduces pro-inflammatory cytokine, IL-10, and ICAM-1 mRNA expression compared to normothermia. Inhibition of adhesion molecule expression and microglial activation has also been confirmed by Deng and colleagues in rat models of both focal cerebral ischemia and brain inflammation [35]. Thus, the beneficial effects of hypothermia on neuroprotection are considered to be due, in part, to suppression of post-injury pro-inflammatory factors by microglia. However, the role of hypothermia in modulating anti-inflammatory cytokines, for example, IL-10, remains controversial. While mild hypothermia has been shown to increase plasma IL-10 concentration in endotoxemic rats, thus potentially mediating the anti-inflammatory effects of hypothermia [36,37], Matsui et al. [38] and Russwurm et al. [39] have previously demonstrated that mild hypothermia inhibits IL-10 production in peripheral blood mononuclear cells. Interestingly, in lipopolysaccharide-activated cultured microglia cells isolated from rats, hypothermia has also been found to reduce production of IL-6, IL-10, and nitric oxide, suggesting that the neuroprotective effects of hypothermia might involve not only the inhibition of pro-inflammatory factors, but also the inhibition of anti-inflammatory factor(s) [40]. Comparably, we found less upregulation of IL-10 mRNA expression in the cerebral tissue in the hypothermia group compared to normothermia after successful CPR.

Since IL-1β was one of the cytokines that was strongly up-regulated on mRNA level in our study, we decided to evaluate IL-1β expression also on the protein level. Analysis of cerebral cortex tissue using a swine specific ELISA system revealed significantly increased IL-1β protein concentration compared with the sham control group after cardiac arrest and normothermia but not after hypothermia. Interestingly, Callaway et al. have recently demonstrated that hypothermia after cardiac arrest did not alter serum inflammatory markers, suggesting that circulating cytokines may not play a specific role regarding the neuroprotective effect of hypothermia [25]. In contrast, it is well conceivable, that local cerebral cytokines released by brain cells will affect more extensively various cerebral ischemia/reperfusion injury cascades and will have a much broader effect on brain damage than systemically elevated levels of cytokines.

Concerning reliable biochemical markers of brain tissue damage, increased serum levels of the low molecular weight protein S100B have been reported after cardiac arrest correlating with neurological complications. However, mild therapeutic hypothermia did not affect S100B serum levels in survivors of cardiac arrest in several clinical studies [41,42]. In addition, Xiao and colleagues have previously shown that cardiac arrest significantly increased brain myeloperoxidase activity, but again, mild hypothermia had no effect. Thus, the hypothermia-elicited neuroprotection seemed not to be neutrophil-dependent, at least in that rat model of asphyxial cardiac arrest [43].

### Effects of pharmacological post-conditioning on cerebral inflammatory response

Volatile anesthetic agents have emerged as pre-conditioning-like agents with significant neuroprotective effects and the ability to reduce excitotoxic induced cell death, to decrease cerebral metabolic rate, to activate inducible nitrous oxide synthase and p38 mitogen-activated protein kinases, and to improve neurological deficits in models of both focal and global cerebral ischemia [6,44,45]. Most experimental studies have documented improved functional performance when neuroprotective agents were given before the insult. In patients with cardiac arrest, however, pretreatment is virtually impossible because of the unpredictable onset of ischemia. Therefore, as in our study, potential protective interventions should be initiated during or after experimental ischemia to affect reperfusion injury. In this context, pharmacological postconditioning with volatile anesthetics in addition to mild hypothermia may offer an attractive opportunity to further ameliorate brain damage and inflammation in the post-resuscitation period. The effects of volatile agents on the inflammatory response after cardiac arrest have not yet been elucidated. In endotoxemic rats, inhalation of sevoflurane significantly attenuated plasma levels of TNFα and IL-1β [46]. In addition, sevoflurane post-conditioning showed anti-inflammatory and anti-necrotic effects in cultured kidney proximal tubule cells [47], and sevoflurane attenuated the inflammatory response upon stimulation of alveolar macrophages with endotoxin in vitro [48]. In our study, however, sevoflurane administered instead of propofol during reperfusion after successful CPR did not further attenuate local cerebral inflammatory response. These observations are comparable to those obtained in a study by Fries et al. where the volatile anesthetic isoflurane did not reduce neurological dysfunction and histopathological alterations induced by cardiac arrest [49]. However, it is conceivable that hypothermia alone has such potent anti-inflammatory properties compared to normothermia, that an additional effect of sevoflurane could not be revealed in the present study. Moreover, potential protective effects of volatile anesthetics depend on energy-dependent signal transduction, for example, protein synthesis and phosphorylation [50], that may be affected by hypothermia-induced decrease of metabolic rate as well as suppression of protein synthesis.

### Cerebral apoptosis-related mRNA expression

In the cerebral cortex tissue, we found a significant upregulation of both Bcl-2 and Bax mRNA expression after global cerebral ischemia. Comparably, Mishra and colleagues have recently reported increased apoptosis in a pig model of cerebral hypoxia for 60 minutes, indicated by an increased ratio of Bax/Bcl-2 protein concentration, activation of caspase-9, lipid peroxidation, and DNA fragmentation in mitochondria of the cerebral cortex [51].

Besides the regulation of inflammatory molecules, mild therapeutic hypothermia significantly attenuated the mRNA expression of the apoptosis-regulating proteins Bax and Bcl-2 in our study. These results are partly comparable to the findings of Eberspächer et al. [52,53], where hypothermia prevented an ischemia-induced increase of the pro-apoptotic protein Bax, but did not change or even increase expression of the anti-apoptotic protein Bcl-2. Potential discrepancies between the work presented here and those in the literature could be due to the type of species and duration of ischemia. In our pig model seven minutes of cardiac arrest were followed by resuscitation compared with the latter studies investigating a rat model of common carotid artery occlusion plus hemorrhagic hypotension [52,53]. In a similar rat model of cerebral ischemia, Pape et al investigated the effects of sevoflurane on neuronal damage and expression of apoptic factors. Sevoflurane was administered before, during and after cerebral ischemia, and has been found to modulate the balance between pro- and anti-apoptotic key proteins towards a reduction of active programmed cell death by increasing the hippocampal concentration of the anti-apoptotic proteins Bcl-2, and by inhibiting the ischemia-induced upregulation of the pro-apoptotic protein Bax [54].

In our pig model of cardiac arrest, however, sevoflurane post-conditioning combined with mild hypothermia did not confer additional effects in terms of apoptotic-related mRNA expression. Again, it is conceivable that hypothermia alone has such potent anti-apoptotic effects, that an additional effect of sevoflurane could not be revealed in the present study.

### Limitations

Although we used a porcine model of cardiac arrest following myocardial ischemia reflecting a common clinical scenario, there are several points that need to be addressed in future studies: (i) both long-term survival and neurological outcome were not evaluated because of limitations posed by governmental regulations; therefore, we did not assess the relationship between the upregulation of cytokines and post-resuscitation cerebral dysfunction. (ii) Blinding the investigator was not possible throughout the experiment due to the cooling technique, but tissue samples were analyzed by a person blinded to treatment assignment.

## Conclusions

In conclusion, (i) global cerebral ischemia following cardiac arrest results in up-regulation of pro-inflammatory cytokines; (ii) hypothermia after cardiac arrest reduces up-regulation of various cytokines in the cerebral tissue. This may promote, at least in part, neuroprotection. (iii) The volatile anesthetic sevoflurane, when administered during reperfusion after successful CPR, did not confer statistically significant additional anti-inflammatory effects in the above setting.

## Key messages

• Global cerebral ischemia following cardiac arrest results in up-regulation of local pro-inflammatory cytokines expression.

• Mild hypothermia after cardiac arrest attenuates cerebral inflammatory response.

• Sevoflurane does not confer additional anti-inflammatory effects.

• Further studies on the relationship between cerebral inflammatory response and post-resuscitation cerebral dysfunction are warranted.

## Abbreviations

BL: baseline; CPR: cardiopulmonary resuscitation; ELISA: enzyme-linked immunosorbent assay; HT: hypothermia; ICAM-1: intercellular adhesion molecule-1; IL: interleukin; LAD: left anterior descending (coronary artery); NT: normothermia; ROSC: return of spontaneous circulation; RT-PCR: reverse transcriptase polymerase chain reaction; SEV: sevoflurane; TIVA: total intravenous anesthesia; TNFα: tumor necrosis factor α; VF: ventricular fibrillation.

## Competing interests

The authors declare that they have no competing interests.

## Authors' contributions

PM, KDZ and BB conceived and designed the experiments. PM, MG, KDZ and MA performed the experiments. MG, MA, RL, NF, JH and KZ analyzed the data. PM, KDZ, MA and BB wrote the paper. All authors read and approved the final manuscript.

## Supplementary Material

Additional file 1 **Extended Method section - Quantitative real-time RT-PCR**. Detailed description of quantitative real-time RT-PCR, primer sequences and TaqMan probes. Click here for file
