# Supplementary material for: Mild hypothermia alone or in combination with anesthetic post-conditioning reduces expression of inflammatory cytokines in the cerebral cortex of pigs after cardiopulmonary resuscitation
Source: Crit Care. 2010 Feb 16;14(1):R21. doi: 10.1186/cc8879 (PMC2875536; doi:10.1186/cc8879)
Supplement: Additional file 1 — Extended Method section - Quantitative real-time RT-PCR. Detailed description of quantitative real-time RT-PCR, primer sequences and TaqMan probes. [file cc8879-S1.doc]

**Additional File 1**

**Title: Extended Method section - Quantitative real-time RT-PCR.**

**Description: Detailed description of quantitative real-time RT-PCR, primer sequences and TaqMan probes**

Transcript levels of interleukin (IL)-1β, IL-6, IL-10, tumor necrosis factor (TNF)α, intercellular adhesion molecule (ICAM)-1, and the apoptosis-associated proteins Bax and Bcl-2 were investigated in the cerebral cortex tissue of all surviving animals and compared with tissue of sham control animals.

Gene expression levels were determined using quantitative real-time reverse transcriptase polymerase chain reaction (RT-PCR). Total RNA was isolated from frozen tissue using a commercially available kit (RNeasy Mini Kit; Qiagen, Hilden, Germany). RNA yield and purity were measured by a spectrophotometer (Spectronic GENESYS 10UV, Rochester, USA). 500 ng RNA was subsequently used for the synthesis of first-strand cDNA with random hexamers using the MultiScribe™ Reverse Transcriptase System (Applied Biosystems, Foster City, USA) according to the manufacturer’s instructions. Integrity of RNA was determined by gel electrophoreses on a standard 2% agarose gel stained with ethidium bromide and visualized by exposure to ultraviolet light. Quantitative PCR was performed utilizing the TaqMan Universal PCR Master Mix (Applied Biosystems, Foster City, USA). Genes of interest were amplified using custom primers and probes. All reactions were performed on the ABI Prism 7900HT Sequence Detection System (Applied Biosystems, Foster City, USA) utilizing the following conditions: Stage 1, 2 minutes at 50°C; stage 2, 10 minutes at 95°C; stage 3, 45 cycles of 15 seconds of melting at 95°C followed by DNA synthesis for 1 minute at 60°C. Stable standard gene expression for internal standardization of target gene expression data was determined by BestKeeper application [17]. Out of three candidates, β-actin, HPRT, and glyceraldehyde-3-phosphate dehydrogenase (GAPDH), GAPDH demonstrated least variations. Thus, all samples were normalized for input based on GAPDH. Intron spanning primers and minor groove binder probes used for quantitative RT-PCR were purchased as Assay-on-Demand from Applied Biosystems (Nieuwekerk a/d IJsel, The Netherlands). Primers were designed using primer design software [18]. Primers were analyzed using Oligo Analysis & Plotting Tools software from Operon, in order to avoid secondary structures such as hairpins and loops. Primer sequences and amplicon lengths are shown in table S1. Serial cDNA dilution curves were produced to calculate the amplification efficiency for all genes. A graph of threshold cycle (Ct) versus log10 relative copy number of the sample from a dilution series was produced. The slope of the curve was used to determine the amplification efficiency: efficiency = 10 (-1/slope) [19].

Data analysis was performed according to a relative standard curve method using an Excel spreadsheet, and statistical significance was tested using randomization testing, as provided in the REST2005 program [20]. Samples with a probability value of < 0.05 were regarded to be significant different between groups. The ratio of the target gene expression level was determined for each sample, and the results are expressed as x-fold increase over sham.

**Table S**1. Primer sequences and TaqMan probes.

| Target | Forward | Backward | TaqMan | Reporter | Quencher |
| --- | --- | --- | --- | --- | --- |
| GAPDH | attgccctcaacgaccact | ggcctctctcctcctcgc | tccaccaccctgttgctgtagccaaat | FAM | TAMRA |
| HPRT | cggcctccgttatggcg | ggtcataacctggttcgtcatca | cgcagccccagcgtcgtgatta | FAM | TAMRA |
| β-Aktin | tcatcaccatcggcaacg | ttcctgatgtccacgtcgc | ccttcctgggcatggagtcctgc | FAM | TAMRA |
| IL-1β | atgctgaaggctctccacct | ttgttgctatcatctccttgcac | aagctcatgcagaacaccacttctctcttcaagt | FAM | TAMRA |
| IL-6 | gaactccctctccacaagcg | gggtagggaaggcagtagcc | cttcagtccagtcgccttctccctgg | FAM | TAMRA |
| IL-10 | gctggaggactttaagggttacc | atatcctcccatcactctctgc | ttgccaagccttgtcagagatgatccag | FAM | TAMRA |
| TNFα | ggcccaaggactcagatca | cggctttgacattggctaca | caaacctcagataagcccgtcgcc | FAM | BBQ |
| ICAM-1 | ctggcagacgagaaggtggt | gctcgctcagggtcaggtt | tgaccttctacagcttcccacctccca | FAM | TAMRA |
| Bax | gcattggagatgaactggacagtaac | cagtttactggcaaagtagaaaagc | acggactccccccgagaagtctttt | FAM | TAMRA |
| Bcl-2 | ttgccgagatgtccagcc | tcccccagttcaccccat | cctgaagagctcctccaccaccgt | FAM | TAMRA |

Primer sequences and TaqMan probes of the interleukin (IL)-1β, IL-6, IL-10, tumor necrosis factor (TNF)α, intercellular adhesion molecule (ICAM)-1, Bax, and Bcl-2. GAPDH indicates glyceraldehyde-3-phosphate dehydrogenase ; HPRT, hypoxanthin-guanin-phosphoribosyltransferase.
